# Evolution of two bulk-superconducting phases in Sr_0.5_*RE*_0.5_FBiS_2_ (*RE*: La, Ce, Pr, Nd, Sm) by external hydrostatic pressure effect

**DOI:** 10.1038/s41598-020-69889-w

**Published:** 2020-07-30

**Authors:** Aichi Yamashita, Rajveer Jha, Yosuke Goto, Akira Miura, Chikako Moriyoshi, Yoshihiro Kuroiwa, Chizuru Kawashima, Kouhei Ishida, Hiroki Takahashi, Yoshikazu Mizuguchi

**Affiliations:** 1 Department of Physics, Tokyo Metropolitan University, 1-1 Minami-Osawa, Hachioji, Tokyo 192-0397 Japan; 2 Faculty of Engineering, Hokkaido University, Kita-13, Nishi-8, Kita-ku, Sapporo, Hokkaido 060-8628 Japan; 3 Graduate School of Advanced Science and Engineering, Hiroshima University, 1-3-1 Kagamiyama, Higashihiroshima, Hiroshima 739-8526 Japan; 4 Department of Physics, College of Humanities and Sciences, Nihon University, Setagaya, Tokyo 156-8550 Japan

**Keywords:** Electronic properties and materials, Superconducting properties and materials

## Abstract

Polycrystalline samples of Sr_0.5_*RE*_0.5_FBi*S*_2_ (*RE*: La, Ce, Pr, Nd, and Sm) were synthesized via the solid-state reaction and characterized using synchrotron X-ray diffraction. Although all the Sr_0.5_*RE*_0.5_FBiS_2_ samples exhibited superconductivity at transition temperatures (*T*_c_) within the range of 2.1–2.7 K under ambient pressure, the estimated superconducting volume fraction was small, which indicates non-bulk nature of superconductivity in those samples under ambient pressure. A dramatic increase in shielding fraction, which indicates the emergence of the bulk superconductivity was achieved by applying external hydrostatic pressures. We found that two phases, low-*P* phases with *T*_c_ = 2.5–2.8 K and high-*P* phases with *T*_c_ = 10.0–10.8 K, were induced by the pressure effect for samples with RE = La, Ce, Pr, and Nd. Pressure-*T*_c_ phase diagrams indicated that the critical pressure for the emergence of the high-*P* phase tends to increase with decreasing ionic radius of the doped *RE* ions, which was explained by the correlation between external and chemical pressure effects. According to the high-pressure X-ray diffraction measurements of Sr_0.5_La_0.5_FBiS_2_, a structural phase transition from tetragonal to monoclinic also occurred at approximately 1.1 GPa. Bulk superconducting phases in Sr_0.5_*RE*_0.5_FBiS_2_ induced by the external hydrostatic pressure effect are expected to be useful for understanding the effects of both external and chemical pressures to the emergence of bulk superconductivity and pairing mechanisms in Bi*Ch*_2_-based superconductors.

## Introduction

The discovery of superconductivity in BiS_2_-based compounds^1,2^ such as Bi_4_O_4_S_3_ and *RE*O_1-*x*_F_*x*_BiS_2_ has triggered numerous studies focusing on identifying new superconductors featuring a higher transition temperatures (*T*_c_) and elucidating the mechanisms of superconductivity in BiS_2_-based compounds. Due to the similarity of the crystal structure among the BiS_2_-based, cuprates and iron-based compounds^3,4^, BiS_2_-based compounds have been considered as a new example of layered superconductor family. Thus far, various types of BiS_2_-based compounds have been synthesized by replacing different blocking layers^1,2,5–17^, such as the Bi_4_O_4_(SO_4_)_1−*x*_ layer, the *RE*O layer (*RE*: La, Ce, Pr, Nd, and Sm), or the *AE*F layer (*AE*: Sr or Eu). Notably, some BiS_2_-based compounds do not exhibit bulk superconductivity even after electron doping. To induce bulk superconductivity in BiS_2_-based compounds, various chemical substitutions have been attempted (see review articles)^14,18^. Among these, the iso-valent substitutions, such as Nd^3+^ substitutions for La^3+^ or Se^2−^ substitutions for S^2−^, were found to be effective for inducing bulk superconductivity. Based on systematic structural analyses, we have revealed that the in-plane chemical pressure is one of the essential parameters that facilitate the emergence of bulk superconductivity in Bi*Ch*_2_-based (*Ch*: S, Se) systems^18,19^. By applying in-plane chemical pressure, the local structural disorder in the tetragonal structure is removed, and bulk superconductivity is induced^20,21^. To induce bulk superconductivity, external pressure effects are also effective^22–32^. For REO_0.5_F_0.5_BiS_2_ and EuFBiS_2_, the *T*_c_ at ambient condition (~ 2.5 K and ~ 0.5 K) dramatically increase to ~ 10 K under high pressure^23–26^, and the origin of the increase in *T*_c_ was explained by a structural transition from tetragonal (*P*4/*nmm*) to monoclinic (*P*2_1_/*m*) at around 1 GPa for LaO_0.5_F_0.5_BiS_2_ and EuFBiS_2_^23,24^. In spite of the interesting phenomena under high pressure in those BiS_2_-based compounds, the pressure studies have been performed by electrical resistivity measurements. To the best of our knowledge, only two works have used magnetization as a probe for the pressure studies, which confirmed bulk superconductivity in LaO_0.5_F_0.5_BiS_2_ and EuFBiS_2_ under high pressure^23,24^. Therefore, further experiments on how the bulk characteristics of superconductivity could be achieved under pressures are needed. In addition, as summarized above, there are two ways, chemical and external pressure effects, to induce bulk superconductivity. Note that those two bulk superconducting phases have a different crystal structure system of tetragonal (under chemical pressure) and monoclinic (under external pressure). Therefore, in order to further investigate the interplay among superconducting characteristics (*T*_c_ and bulk nature), external pressure, and chemical pressure, it is essential to establish a new system where discussion about the relationships among those factors is possible. In this study, to search for a system which enables us to study such relationships, we have studied the chemical and external pressure effects for the Sr_0.5_*RE*_0.5_FBiS_2_ (*RE*: La, Ce, Pr, Nd, and Sm) system.

SrFBiS_2_ is a parent phase of those target materials and a semiconductor with a band gap. The substitution of Sr^2+^ with *RE*^3+^ induces electron carriers in the BiS_2_ layer, and filamentary superconductivity appears at approximately 2.8 K in La-, Ce-, and Pr-doped compounds^12–15,29–32^. Based on electrical resistivity measurements conducted under high pressures, a considerable increase in *T*_c_ was observed in previous pressure experiments^22,29–32^. The highest *T*_c_ in Sr_0.5_*RE*_0.5_FBiS_2_ is approximately 10 K, and the pressure dependences of *T*_c_ exhibits a sharp increase at the critical pressure (~ 1 GPa). Since there has been no further report on the pressure-induced superconductivity in Sr_0.5_*RE*_0.5_FBiS_2_, we have studied the superconducting phase diagrams for Sr_0.5_*RE*_0.5_FBiS_2_ from magnetization experiments under high pressure and discussed the interplay among superconducting characteristics, chemical pressure, and external pressure. Two bulk superconducting phases with a lower *T*_c_ (low-*P* phase) and a higher *T*_c_ (high-*P* phase) were confirmed.

## Results

### Sample characterization and physical properties at ambient pressure

**Figure 1:**
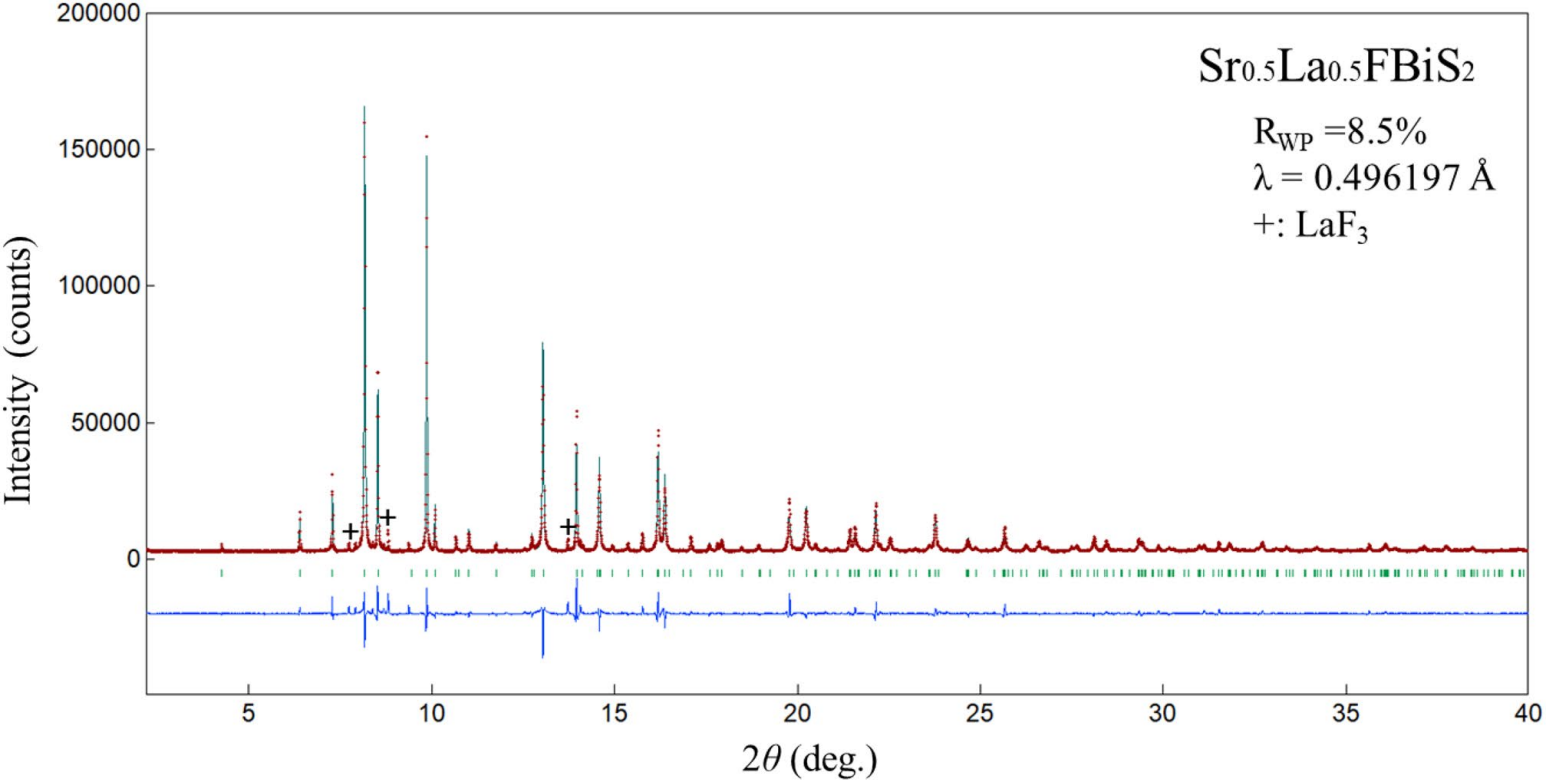
Synchrotron powder XRD patterns for Sr_0.5_La_0.5_FBiS_2_. Symbol of + indicates the impurity of LaF_3_.

**Figure 2:**
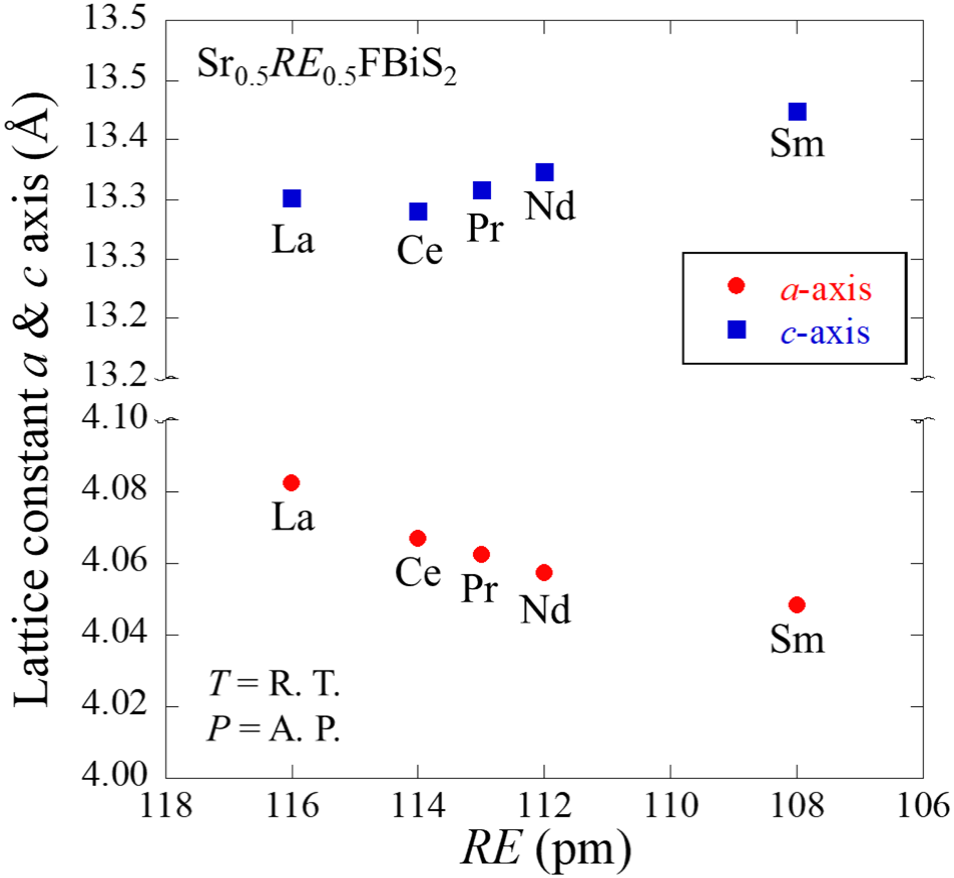
Dependences of the lattice constants *a* and *c* as a function of the *RE*^3+^ (*RE*: La, Ce, Pr, Nd, and Sm) ionic radius.

**Figure 3:**
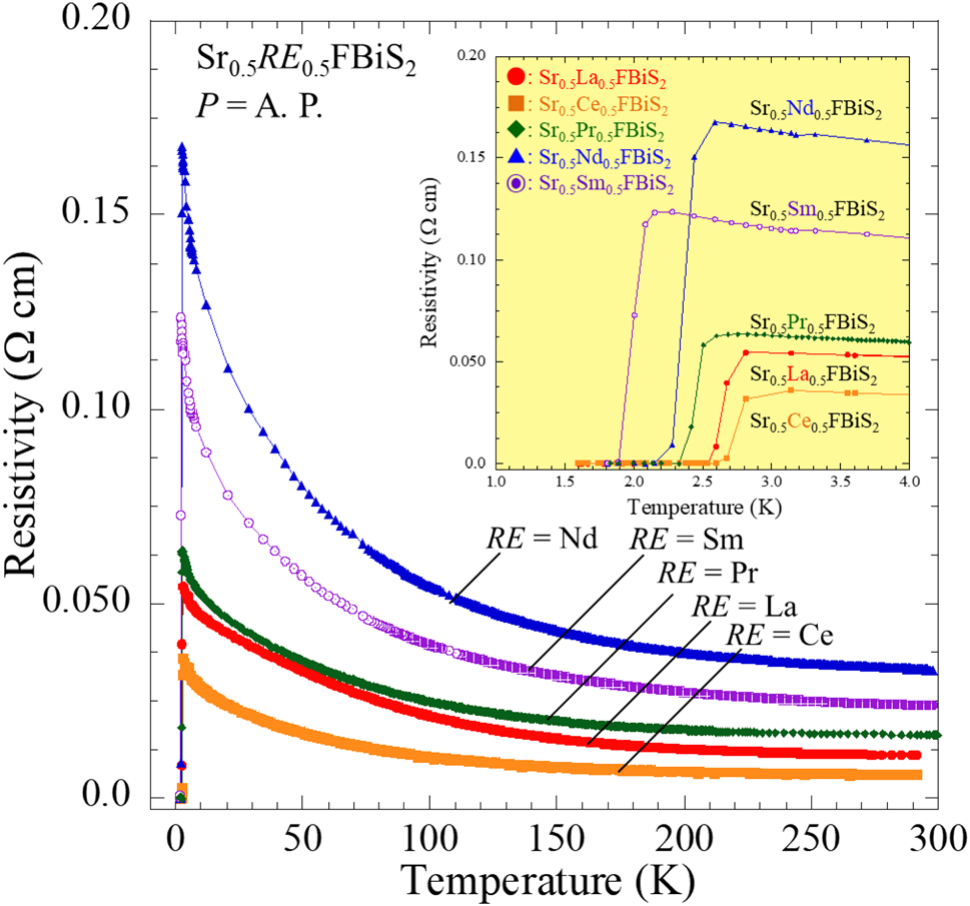
Temperature dependence of resistivity for Sr_0.5_*RE*_0.5_FBiS_2_ (*RE*: La, Ce, Pr, Nd, and Sm) at ambient pressure. A. P. denotes an ambient pressure condition.

**Figure 4:**
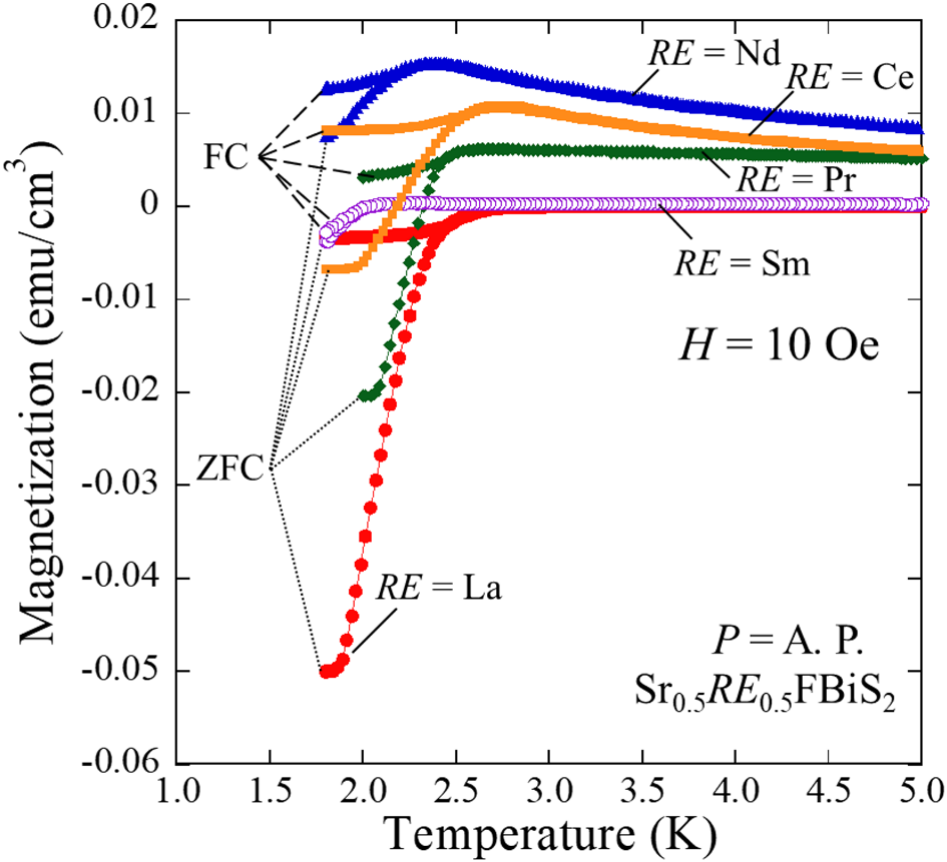
Temperature dependence of magnetization for Sr_0.5_*RE*_0.5_FBiS_2_ (*RE*: La, Ce, Pr, Nd, and Sm) at ambient pressure. Dashed and dotted lines indicate a field cooling (FC) and zero-field cooling (ZFC), respectively. A. P. denotes an ambient pressure condition.

Figure 1 depicts the powder synchrotron X-ray diffraction (XRD) patterns for Sr_0.5_La_0.5_FBiS_2_. For Sr_1-*x*_*RE*_*x*_FBiS_2_ (*RE* = Ce, Pr, Nd, and Sm), see Supplemental Fig. S1a–d. The crystal structure of the obtained samples was well refined using the Rietveld method. They were well refined using a tetragonal structure with the *P*4/*nmm* space group. Small impurity peaks due to *RE*F_3_ (*RE*: La, Ce) and Bi_2_S_3_ were also detected in the case of the Pr-, Nd-, and Sm-based samples. As shown in Fig. 2, we observed that the lattice constant *a* decreased with decreasing *RE* ionic radius; however, the lattice constant *c* increased under these conditions. The obtained values were in agreement with previous reports^12,30,31^. The chemical composition ratios of the samples were determined using energy dispersive X-ray spectroscopy (EDX). These results showed that the chemical compositions of the obtained samples were in reasonable agreement with the nominal compositions (Table 1). The electrical resistivity of the samples at ambient pressure was measured down to 1.6 K (Fig. 3). In all the samples, semiconducting behaviour was observed; moreover, the resistivity-temperature (*ρ*-*T*) curve indicated a slight increase in *ρ* on cooling, which implied that the conduction electrons were weakly localized due to the in-plane local disorder in the BiS_2_ layer^18–21^. A superconducting transition was observed at *T*_c_ = 2.7, 2.7, 2.6, 2.6, and 2.1 K for *RE* = La, Ce, Pr, Nd, and Sm, respectively (see the inset of Fig. 3). In addition, a superconducting transition was also observed in the temperature dependence of magnetization, as plotted in Fig. 4. The *T*_*c*_ estimated based on magnetization was in agreement with that obtained based on the resistivity measurements. This is the first study to report on the observation of superconductivity in Sr_0.5_Nd_0.5_FBiS_2_ and Sr_0.5_Sm_0.5_FBiS_2_ under ambient pressure. However, in these samples, the estimated shielding volume fraction was less than 6%. This indicates that chemical pressure effects, which is expected to be generated by the substitution of smaller *RE* such as Nd and Sm, are insufficient to induce bulk superconductivity in the Sr_1−*x*_*RE*_*x*_FBiS_2_ system. To obtain bulk superconductivity, which is essentially important for describing intrinsic superconducting properties, we applied external pressure on the samples.

**Table 1:** Actual composition (mol%) from the EDX analysis against the nominal composition (mol%). Fluorine amount is regarded as 1.

Nominal composition	Actual composition
Sr_0.50_La_0.50_FBiS_2_	Sr_0.52_La_0.48_FBi_1.00_S_2.00_
Sr_0.50_Ce_0.50_FBiS_2_	Sr_0.54_Ce_0.48_FBi_0.98_S_2.00_
Sr_0.50_Pr_0.50_FBiS_2_	Sr_0.56_Pr_0.41_FBi_0.99_S_2.04_
Sr_0.50_Nd_0.50_FBiS_2_	Sr_0.60_Nd_0.39_FBi_0.98_S_2.03_
Sr_0.50_Sm_0.50_FBiS_2_	Sr_0.64_Sm_0.39_FBi_0.97_S_2.01_

### External pressure effect

**Figure 5:**
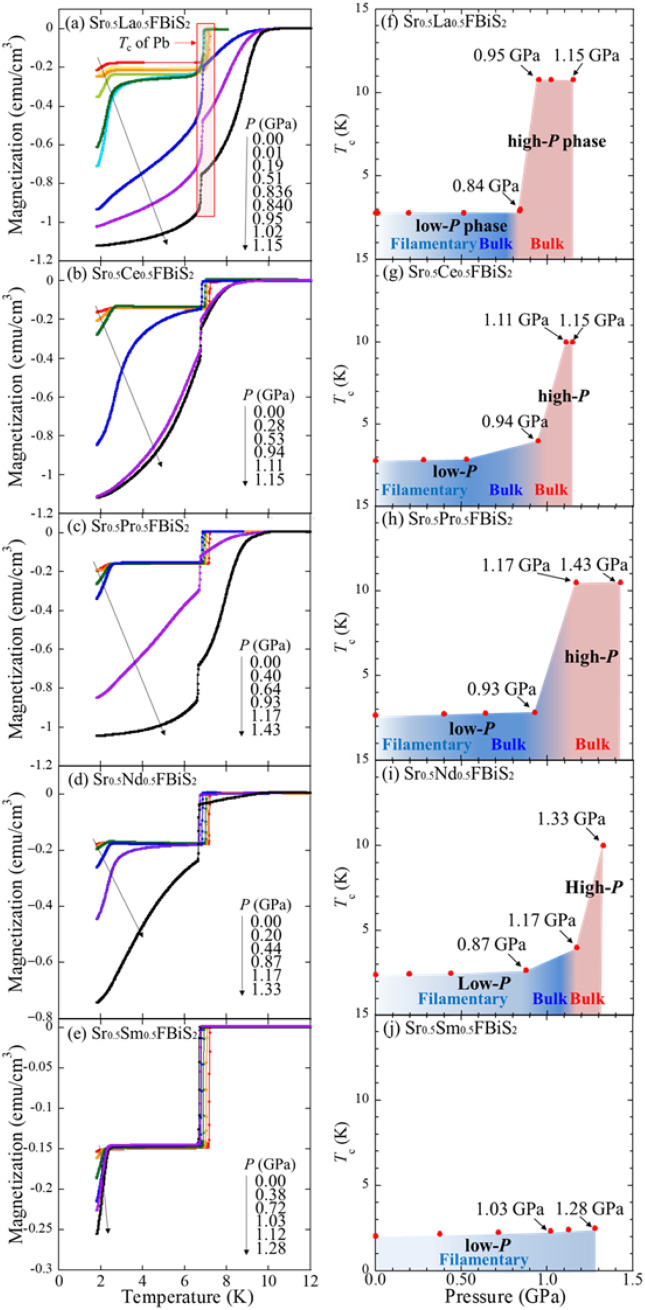
(**a**–**e**) Temperature dependences of magnetization under various pressure (*P*_La_ = 0, 0.01, 0.19, 0.51, 0.836, 0.840, 0.95, 1.02, and 1.15 GPa; *P*_Ce_ = 0, 0.28, 0. 53 0.94, 1.11, and 1.15 GPa; *P*_Pr_ = 0.0, 0.40, 0.64, 0.93, 1.17, and 1.43 GPa; *P*_Ndr_ = 0.0, 0.20, 0.44, 0.87, 1.17, and 1.33 GPa; *P*_Sm_ = 0.0, 0.375, 0.716, 1.03, 1.12, and 1.28 GPa) for Sr_0.5_*RE*_0.5_FBiS_2_ (*RE*: La, Ce, Pr, Nd, and Sm), (**f**–**j**) pressure dependence of *T*_c_ for Sr_0.5_*RE*_0.5_FBiS_2_ (*RE*: La, Ce, Pr, Nd, and Sm) when a magnetic field of 10 Oe was applied for all samples. The superconducting transition around 7 K indicates the superconducting transition of Pb manometer.

Figure 5a shows the temperature dependences of the magnetization of Sr_0.5_La_0.5_FBiS_2_ when increasing the applied pressure to 1.15 GPa. The *T*_c_ of approximately 2.7 K (low-*P* phase) remained almost unchanged up to 0.84 GPa; alternatively, there was an evident increase in the shielding volume fraction. This result indicates that the external pressure effectively enhances the shielding volume fraction, which corresponds to the emergence of bulk nature of the superconducting states in the Sr_0.5_La_0.5_FBiS_2_ samples at a low-*P* regime. A remarkable increase in *T*_c_ up to *T*_*c*_^max^ = 10.8 K (high-*P* phase) was observed at *P* > 0.95 GPa. The enhancement in the shielding volume fraction was also observed in the high-*P* phase, which was achieved by increasing applied pressure to the maximum pressure, without a noticeable change in the *T*_c_. The drastic increase in *T*_c_ is explained by a tetragonal to a monoclinic phase.

**Figure 6:**
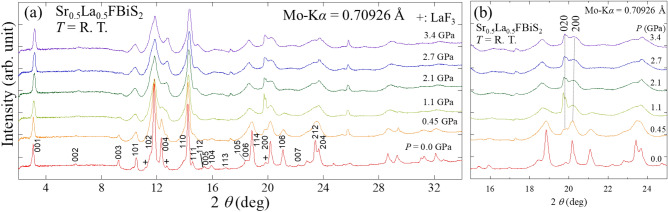
(**a**) XRD patterns (Mo K_α_) of Sr_0.5_La_0.5_FBiS_2_ under various pressure at room temperature. (**b**) Zoomed XRD patterns near the 020 and 200 peaks.

Figure 6a presents the laboratory X-ray diffraction patterns of Sr_0.5_La_0.5_FBiS_2_ at room temperature under various applied pressures of up to 3.4 GPa. Shifts of the (001) and (004) peaks to higher angles clearly indicate the shrinkage of the lattice along the *c*-axis due to the pressure. In contrast, a relatively smaller shift of the (110) peak was detected, which indicates that the in-plane size remains almost unchanged. Strong peak broadening was observed for the (200) peak above 1.1 GPa, indicating peak splitting due to the lowering of in-plane structural symmetry. Similar peak splitting on the (200) peak was observed for isostructural LaO_0.5_F_0.5_BiS_2_ and EuFBiS_2_ samples under high pressure^23,24^, and a resultant structural transition from a tetragonal to monoclinic phase was detected. We noticed that the (200) peak asymmetrically split into two or more peaks, as depicted in Fig. 6b. This unexpected evolutions of the XRD pattern may be due to the inhomogeneity of applied pressure and the flexible nature of the in-plane structure of BiS_2_-based compounds. The critical pressure of 1.1 GPa estimated from the XRD corresponds satisfactorily with the *P*_*c*_ estimated from the magnetization measurements. To further analyse crystal structure of this phase under pressure, synchrotron XRD experiments under homogeneous pressure conditions are needed.

The pressure dependence of *T*_c_ is summarized in Fig. 5f; the light blue, blue, and pink regions indicate the filamentary superconductivity, bulk superconductivity in the low-*P* phase, and bulk superconductivity in the high-*P* phase, respectively. The low-*P* phase shifts to the high-*P* phase when a pressure slightly exceeding the critical pressure (*P*_c_) for the high-*P* phase is applied. In regard to this, a phase diagram was created using the shielding volume fraction of 20% or more as a bulk state in order to discuss the phase transition. In the pressure range of 0–0.84 GPa, *T*_c_(*P*) remains almost constant (~ 2.7 K). The bulk superconductivity of the sample was induced by the pressure for the low-*P* phase region; eventually, the high-*P* phase region emerged at approximately *P* = 0.95 GPa with the bulk superconducting states. The *T*_c_(*P*) for the high-*P* phase region is almost constant (10.8 K).

The temperature dependence of magnetization under pressure and the pressure dependence of *T*_c_ phases for all the samples with different *RE* are summarized in Fig. 5a–j and respectively. The high-*P* phase of Sr_0.5_Sm_0.5_FBiS_2_ was not observed up to 1.28 GPa, which is nearly the upper limit of the pressure measurement apparatus used in this experiment. This is the first report offering evidence of the bulk nature of two different (low-*P* and high-*P*) phases of the Sr_1-*x*_*RE*_*x*_FBiS_2_ (*RE*: La, Ce, Pr, and Nd) superconductors when subjected to pressure.

## Discussion

In this section, we discuss the relationship between external pressure effects, chemical pressure effects, and evolution of superconductivity in Sr_0.5_*RE*_0.5_FBiS_2_. On comparing the evolutions of superconductivity for *RE* = Ce–Sm and that for *RE* = La, a slight decrease in *T*_*c*_ for the low-*P* phase was observed with increasing pressure for the *RE* = Ce, Pr, Nd and, Sm samples. Similar trend was observed in a phase diagram of tetragonal phase of Bi*Ch*_2_-based systems examined as a function of chemical pressure^18,19,33^. Moreover, we observed that the *T*_*c*_ of the high-*P* phase showed a trend of decreasing with decreasing *RE* ionic radius. This trend is also common to that observed in high-pressure studies for *RE*O_0.5_F_0.5_BiS_2_ with different *RE*^22^. The *P*_c_ increased with decreasing *RE* ionic radius. Specifically, *P*_c_ was roughly estimated as 0.95, 1.11, 1.17, and 1.33 GPa for La^3+^ (with an ionic radius of 1.16 Å), Ce^3+^ (with an ionic radius of 1.14 Å), Pr^3+^ (with an ionic radius of 1.13 Å), and Nd^3+^ (with an ionic radius of 1.11 Å), respectively, where those ionic radii are values for a coordination number of 8. We noticed that the shift in *P*_c_ by replacing RE in Sr_0.5_*RE*_0.5_FBiS_2_ is clearly small as compared to the case of *RE*O_0.5_F_0.5_BiS_2_; *P*_c_ was ~ 2 GPa for NdO_0.5_F_0.5_BiS_2_^28^. These trends, the increase in *P*_c_ and the decrease in *T*_*c*_ with a decrease in *RE* ionic radius, were also observed for *RE*O_0.5_F_0.5_BiS_2_ compounds^22,26,28^; specifically, the *T*_c_ varied from approximately 10 to 6 K. The different pressure evolutions of superconducting phases in between the Sr_0.5_*RE*_0.5_FBiS_2_ and *RE*O_0.5_F_0.5_BiS_2_ systems would be understood by the difference in the substitution sites. For Sr_0.5_*RE*_0.5_FBiS_2_, the Sr site is partly (50%) substituted by *RE*, and hence the Sr-F bonds are partly remained. In the case of *RE*O_0.5_F_0.5_BiS_2_, all the *RE* site is replaced by different *RE*, and hence the *RE*-(O,F) bond length should systematically decrease according to the *RE* ionic radius. Therefore, the different sensitivity of the crystal structure and superconductivity to external pressures were observed between Sr_0.5_*RE*_0.5_FBiS_2_ and *RE*O_0.5_F_0.5_BiS_2_. The difference should be caused by the chemical bonding states of the SrF-based and *RE*O-based layers and the interlayer interaction between the blocking layers and BiS_2_ conducting layers. Our results suggest that the structure of the blocking layer largely affects the evolution of superconducting phases under high pressures. The results obtained in this study will be useful for material design of Bi*Ch*_2_-based superconductors with a higher *T*_c_.

## Conclusion

We showed the results of the synthesis, crystal structure analysis, resistivity, and magnetic susceptibility measurements investigated under ambient and high pressures for Sr_0.5_*RE*_0.5_FBiS_2_ (*RE*: La, Ce, Pr, Nd, and Sm). The effects of external pressure on magnetization resulted in abrupt increments in *T*_c_ up to 10–10.8 K for the samples with *RE* = La, Ce, Pr, and Nd. Based on the analyses of the shielding volume fraction estimated via magnetic susceptibility measurements, we found that two bulk superconducting phases (low-*P* and high-*P* phases) can be induced by external pressure for Sr_0.5_*RE*_0.5_FBiS_2_. For *RE* = La, we have confirmed a structural transition from laboratory XRD under high pressure, which is a common trend with those observed for LaO_0.5_F_0.5_BiS_2_ and EuFBiS_2_. The critical pressure, where *T*_c_ sharply increased to the high-*P* phase, shifted to a higher pressure with decreasing *RE* ionic radius. This implied that both the external and chemical pressures were affecting *T*_c_. In addition, we have compared the obtained phase diagrams for Sr_0.5_*RE*_0.5_FBiS_2_ and *RE*O_0.5_F_0.5_BiS_2_. We found differences in the sensitivity of the crystal structure and superconducting characteristics to external pressure effects between Sr_0.5_*RE*_0.5_FBiS_2_ and *RE*O_0.5_F_0.5_BiS_2_, which should be caused by the different chemical bonding states in the blocking layers and the interlayer interaction between the blocking layers and BiS_2_ conducting layers.

## Methods

Polycrystalline Sr_0.5_*RE*_0.5_FBiS (*RE*: La, Ce, Pr, Nd, and Sm) samples were synthesized by solid state reaction method in an evacuated quartz tube. Powders of *RE*_2_S_3_ (*RE*: La (99.9%), Ce (99.9%), Pr (99%), Nd (99%), and Sm (99.9%)), SrF_2_ (99%), Bi (99.999%), and S (99.9999%) were weighed for Sr_0.5_*RE*_*0.5*_FBiS_2_. The mixed powder was subsequently pelletized, sintered in an evacuated quartz tube at 700 °C for 20 h, followed by furnace cooling to room temperature. The obtained compounds were thoroughly mixed and ground, then sintered in the same conditions as the first sintering.

The phase purity and the crystal structure of the Sr_0.5_*RE*_0.5_FBiS_2_ (*RE*: La, Ce, Pr, Nd, and Sm) samples were examined by powder synchrotron XRD (ambient pressure XRD) with an energy of 25 keV (λ = 0.49657 Å) at the beamline BL02B2 of SPring-8 under a proposal No. 2019A1101. The synchrotron XRD experiments were performed at room temperature with a sample rotator system, and the diffraction data were collected using a high-resolution one-dimensional semiconductor detector MYTHEN [Multiple mythen system] with a step of 2*θ* = 0.006°. To investigate the evolution of crystal structure of Sr_0.5_La_0.5_FBiS_2_, laboratory XRD experiments under high pressure up to 3.4 GPa were performed at room temperature using a Mo-K_α_ radiation on a Rigaku (MicroMax-007HF) rotating anode generator equipped with a 100 µm collimator. Daphne 7474 was used as a pressure medium.

The crystal structure parameters were refined using the Rietveld method with a RIETAN-FP software^34^. The actual compositions of the obtained samples were analysed using an energy dispersive X-ray spectroscopy (EDX) on TM-3030 (Hitachi).

The temperature dependence of magnetic susceptibility at ambient pressure and under high pressures were measured using a superconducting quantum interference devise (SQUID) with MPMS-3 (Quantum Design). Hydrostatic pressures were generated by the MPMS high pressure Capsule Cell. The sample was immersed in a pressure transmitting medium (Daphene 7373) covered with a Teflon cell. The pressure at low temperature was calibrated from the superconducting transition temperature of Pb manometer. The electrical resistivity was measured on a GM refrigerator system (Made by Axis) using a conventional four-probe method. For the resistivity measurements, gold wires were connected to the samples with a silver paste.

## Supplementary information

Supplementary Information
