# Supplementary material for: Evolution of two bulk-superconducting phases in Sr0.5RE0.5FBiS2 (RE: La, Ce, Pr, Nd, Sm) by external hydrostatic pressure effect
Source: Sci Rep. 2020 Jul 30;10:12880. doi: 10.1038/s41598-020-69889-w (PMC7393496; doi:10.1038/s41598-020-69889-w)
Supplement: Supplementary file 1 — Supplementary Information [file 41598_2020_69889_MOESM1_ESM.docx]

Supplemental Information for “Evolution of two bulk-superconducting phases in Sr_0.5_*RE*_0.5_FBiS_2_ (*RE*: La, Ce, Pr, Nd, Sm) by external hydrostatic pressure effect”

Aichi Yamashita^1^, Rajveer Jha^1^, Yosuke Goto^1^, Akira Miura^2^, Chikako Moriyoshi^3^, Yoshihiro Kuroiwa^3^, Chizuru Kawashima^4^, Kouhei Ishida^4^, Hiroki Takahashi^4^, and Yoshikazu Mizuguchi^1,^*

*^1^Department of Physics, Tokyo Metropolitan University, 1-1 Minami-Osawa, Hachioji, Tokyo, 192-0397, Japan*

*^2^Faculty of Engineering, Hokkaido University, Kita-13, Nishi-8, Kita-ku, Sapporo, Hokkaido 060-8628, Japan*

*^3^ Graduate School of Advanced Science and Engineering, Hiroshima University, 1-3-1 Kagamiyama, Higashihiroshima, Hiroshima 739-8526, Japan*

*^4^Department of Physics, College of Humanities and Sciences, Nihon University, Setagaya, Tokyo 156-8550, Japan*


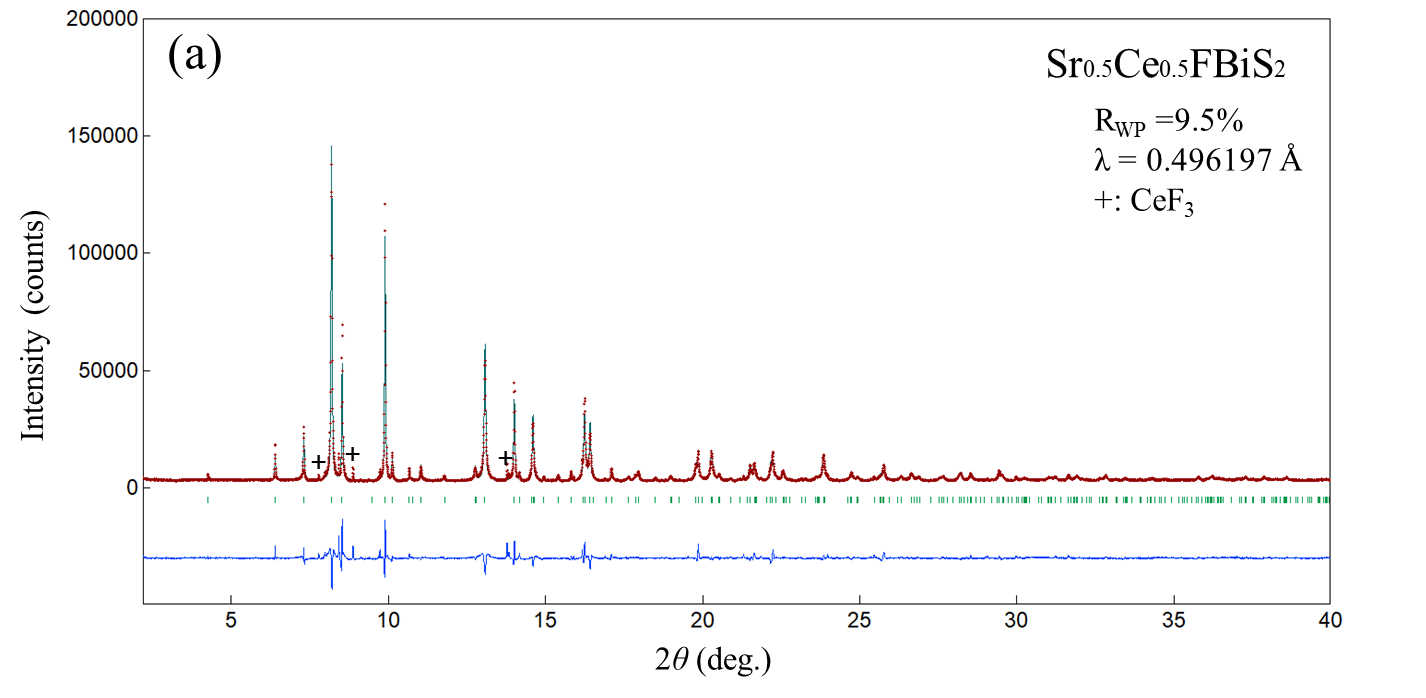


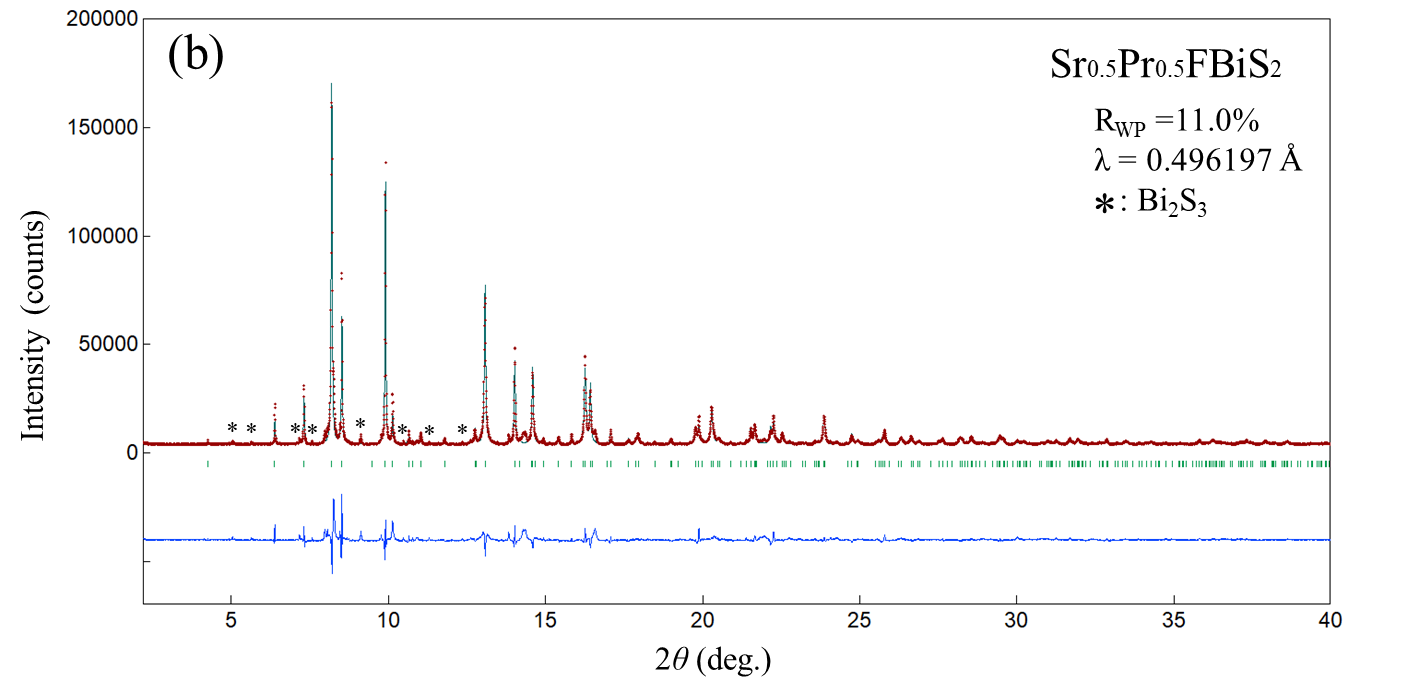

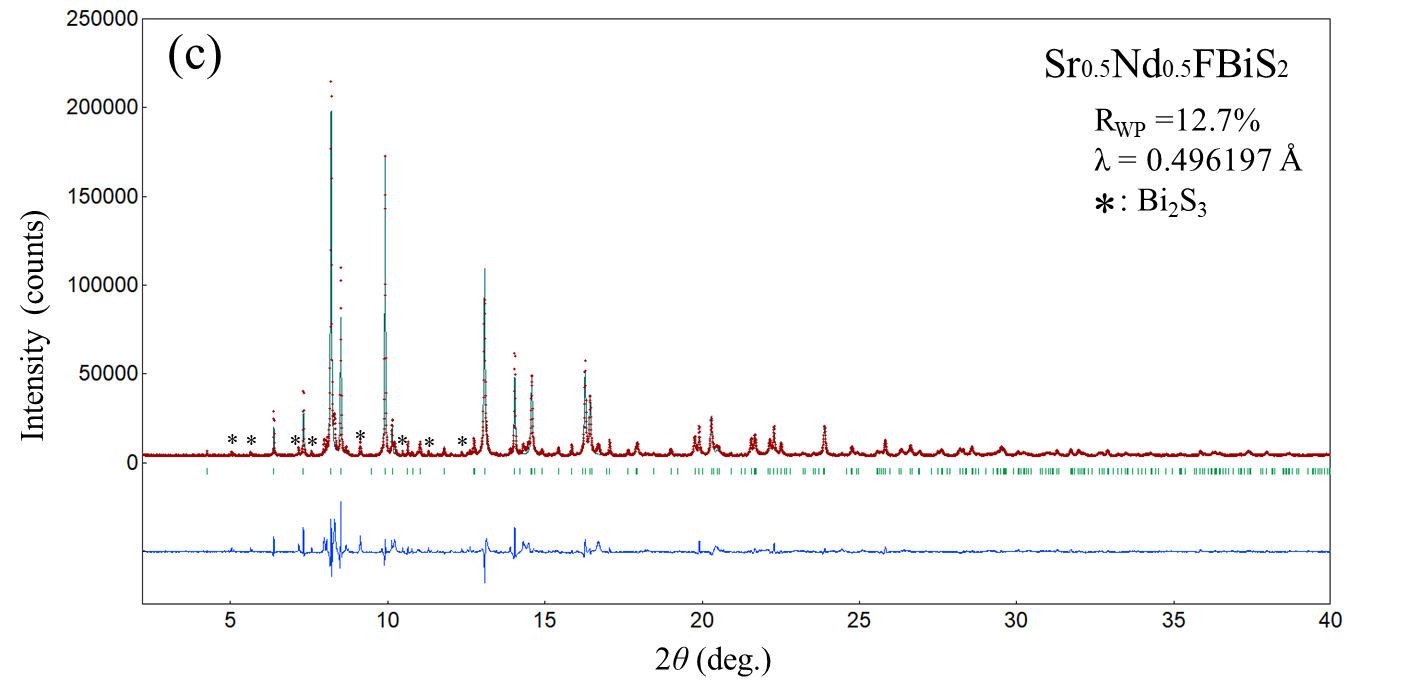


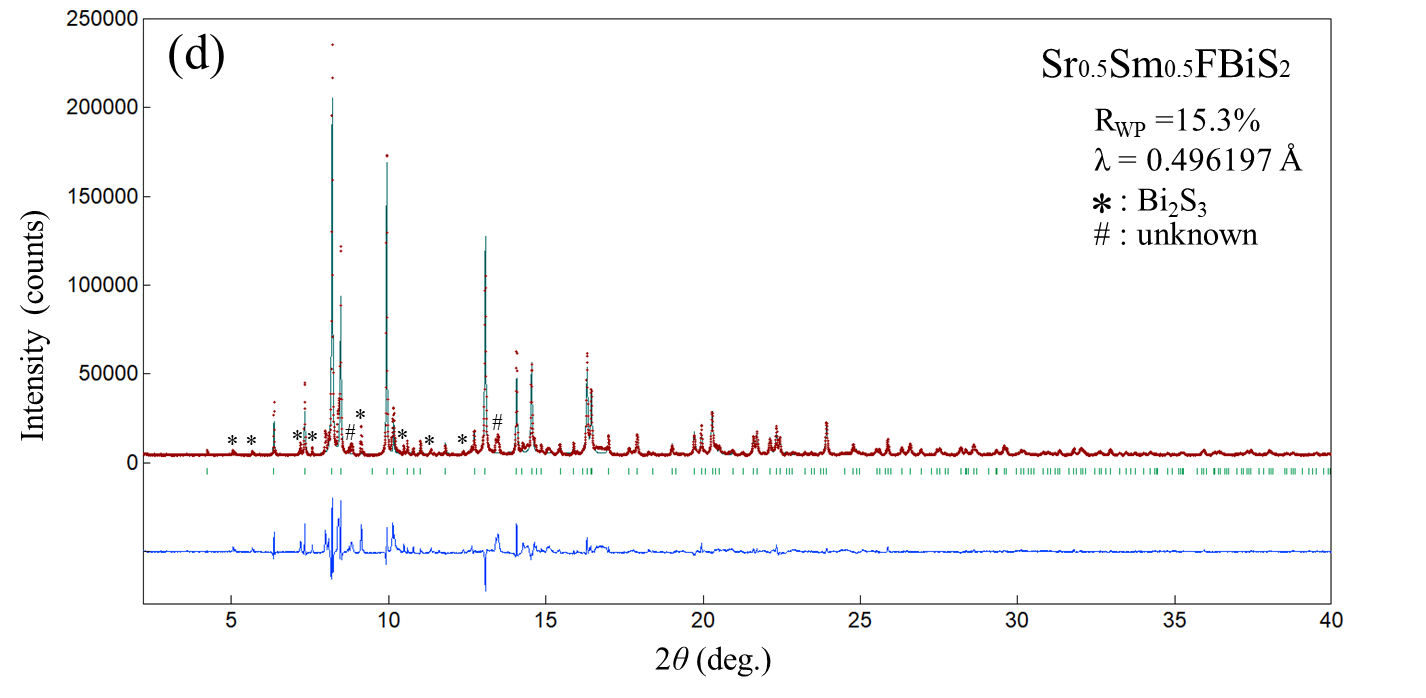


Figure S1 SXRD patterns for Sr_1-_*_x_RE_x_*FBiS_2_. (a) *RE* = Ce, (b) Pr, (c) Nd, and (d) Sm, respectively.

Symbols of +, *, and # indicate the impurities of CeF_3_, Bi_2_S_3_, and unknown, respectively.
